# Experimental Study on Creep Characteristics of Microdefect Articular Cartilages in the Damaged Early Stage

**DOI:** 10.1155/2019/8526436

**Published:** 2019-11-13

**Authors:** Huchen Gong, Yutao Men, Xiuping Yang, Xiaoming Li, Chunqiu Zhang

**Affiliations:** ^1^Tianjin Key Laboratory of the Design and Intelligent Control of the Advanced Mechanical System, Tianjin University of Technology, Tianjin, China; ^2^National Demonstration Center for Experimental Mechanical and Electrical Engineering Education, Tianjin University of Technology, Tianjin, China

## Abstract

Traumatic joint injury is known to cause cartilage deterioration and osteoarthritis. In order to study the mechanical mechanism of damage evolution on articular cartilage, taking the fresh porcine articular cartilage as the experimental samples, the creep experiments of the intact cartilages and the cartilages with different depth defect were carried out by using the noncontact digital image correlation technology. And then, the creep constitutive equations of cartilages were established. The results showed that the creep curves of different layers changed exponentially and were not coincident for the cartilage sample. The defect affected the strain values of the creep curves. The creep behavior of cartilage was dependent on defect depth. The deeper the defect was, the larger the strain value was. The built three-parameter viscoelastic constitutive equation had a good correlation with the experimental results and could predict the creep performance of the articular cartilage. The creep values of the microdefective cartilage in the damaged early stage were different from the diseased articular cartilage. These findings pointed out that defect could accelerate the damage of cartilage. It was helpful to study the mechanical mechanism of damage evolution.

## 1. Introduction

The knee joint is the most complex joint of the human body, which principally play a key role in connecting, bearing, damping, and reducing friction. Because of its high-frequency use and heavy load, it is prone to damage. After cartilage injury, mechanical stress of the joint usually is changed, which causes osteoarthritis. Due to special tissue structure of cartilage, there is no symptom in the early stage of cartilage injury, and it may take several years, ten years, or even decades to develop osteoarthritis. The cartilage has a very poor ability to repair itself, so it might bear the daily load in the state of injury after the cartilage is damaged. Creep experiments are an important method to study the viscoelasticity of solid biomaterials. It describes the history of strain change over time under the constant stress. The creep ability of cartilage determines the bearing capacity. Articular cartilage is a viscoelastic material, and many biomechanical and medical researchers focus on its viscoelastic properties.

Mow et al. [1] carried out the creep and relaxation experiments of bovine articular cartilage and built the linear nonhomogeneous theory to describe the experimental data. Li et al. [2] found by the experiments of bovine articular cartilage that the creep did not affect the relaxation characteristics, but stress relaxation affected creep performance, and relaxation was much faster than creep. Taffetani et al. [3] carried out multiload creep tests using spherical indenters on saturated samples of mature bovine articular cartilage. Ikeuchi et al. [4] found that the creep deformation was related to the contact pressure. Fick and Espino [5] found that the compressive strains of articular cartilage at rupture in the dehydrated degenerate specimens were significantly lower than those measured in the dehydrated healthy tissues. Cooke et al. [6] found that the storage modulus of OA cartilage was shown to be significantly lower than that of healthy cartilage.

Articular cartilage is characterized by the anisotropic and inhomogeneous composition and structure microscopically, which results in its depth-dependent mechanical properties. Chahine et al. [7] found the compression modulus of cartilage to increase with the depth by unconfined compression tests. Gao et al. [8, 9] performed the creep tests with different stress levels and made detailed analysis. They found the Young's modulus of cartilage increased obviously along cartilage depth. The creep strain and creep compliance with creep times decreased from surface to deep. The depth-dependent creep compliance increased with creep time and the increasing amplitude of creep compliance decreased along cartilage depth.

It is well known that damage can alter the mechanical properties of cartilage. It is unclear for mechanistic mechanisms that microinjury cartilage develops osteoarthritis. There are few studies on this problem now. Most researchers did their creep experiments on pathological cartilage or carried out other mechanical experiments on cartilages that had creep deformation [5, 6, 10–13]. However, there are few mechanical experiments on cartilage with defect. Considering that the knee joint of an animal such as a pig is similar to the human knee joint in anatomy, geometry, and function, and obtained easily. In this paper, noncontact digital image correlation technology was used to study creep characteristics of articular cartilages with defect. The influence of defect on creep characteristics of articular cartilage was analyzed and three-parameter creep equation was established. This paper is helpful to understand the mechanical properties of knee cartilage and lay a foundation for researches on the biomechanical mechanism of osteoarthritis formation.

## 2. Experimental Materials and Methods

### 2.1. Experimental Materials

**Figure 1:**
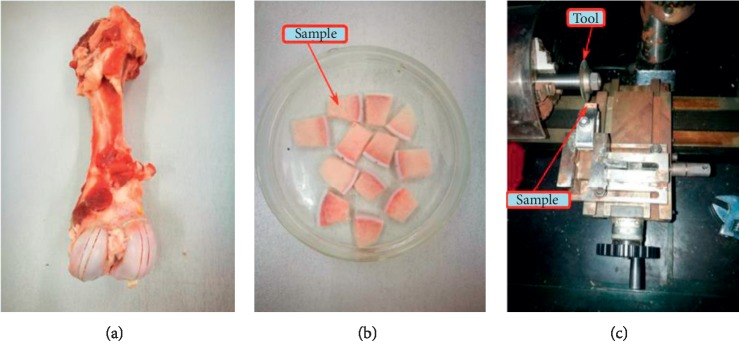
Preparation of cartilage samples: (a) porcine femur; (b) samples; (c) the preparation of defects.

The experimental cartilage samples were taken from the trochlea sites of 8-month-old porcine femurs. The cutting plane is perpendicular to the cartilage surface so that the flat samples can be obtained, which also make compression be distributed on the whole cartilage surface. The cartilage samples with subchondral bone were approximately 10 mm in length and 3 mm in width. The cartilage thicknesses of samples were 1.8–2.2 mm (Figures 1(a) and 1(b)). A micromechanical tool was used to make defects on the cartilage samples (Figure 1(c)). The width of defects was 1 mm and the depth was 0.2 ± 0.02 mm, 0.7 ± 0.02 mm, and 1.1 ± 0.02 mm, respectively. The prepared samples were immersed in physiological saline to remove oil and blood before tests.

Four samples were selected from the prepared samples to operate. To reduce the experimental error, each sample was operated twice. After the first experiment, the sample was immersed in physiological saline for 2 hours so that it can completely rebound to the initial stage, and the same experiment was performed again.

### 2.2. Experimental Equipment and Methods

**Figure 2:**
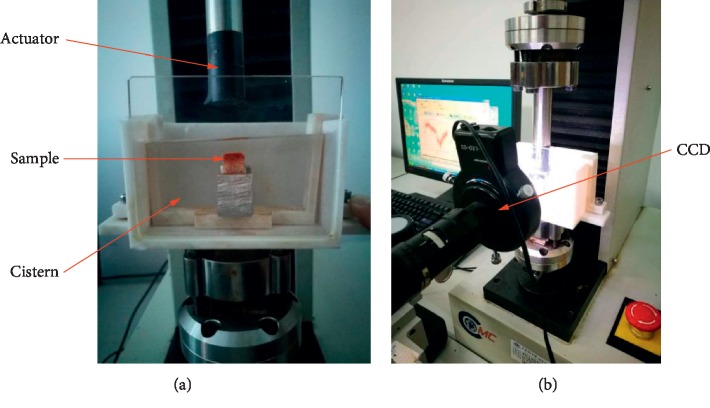
Experimental equipment. (a) Fixture. (b) Fatigue test system and image acquisition system.

All the tests for cartilage samples were carried out on Electronic Universal Fatigue Testing System (EUF-1020), and the digital image correlation (DIC) technique was applied in order to acquire images.  Figure 2 shows the testing apparatus and fixture device.

**Figure 3:**
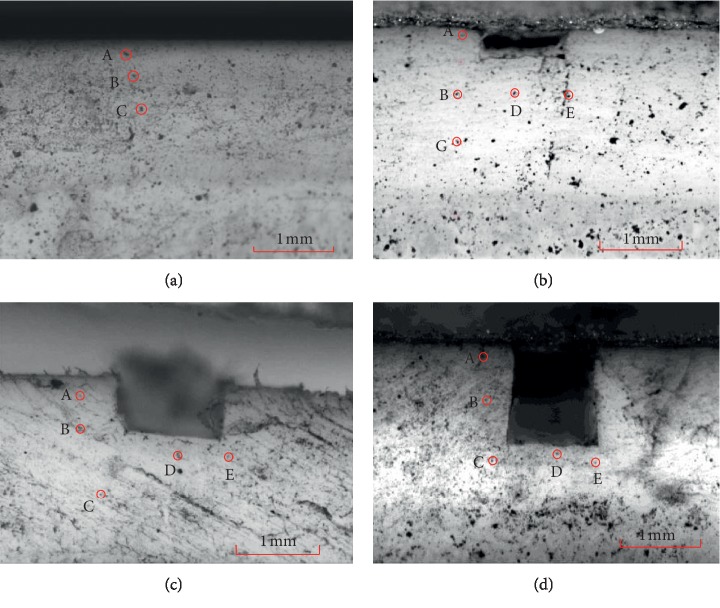
Location of the reference points in different cartilage samples. (a) Intact. (b) Defect depth 0.2 mm. (c) Defect depth 0.7 mm. (d) Defect depth 1.1 mm.

Before the experiment, one profile of the cartilage sample was scattered by iron oxide nanoparticles with a diameter of 10 nm as marks. The sample was fixed on the fixture device and the profile coated mark points faced outside. The physiological saline was added to the water container to prevent the sample from dehydrating and maintain the physiological environment of cartilage during the tests. And then, the experimental parameters of the load machine were set, in which the creep time was 3600 s, the initial load rate was 0.5 MPa/s, and load on the sample was 1 MPa. The image acquisition device was set to capture one picture every 2 seconds and took a total of 2000 pictures. The black spots in the picture were iron oxide nanoparticle groups.  Figure 3 showed the selected reference points of the samples. Points A, B, and C came from the locations of the surface, middle, and deep layers of cartilage, respectively; points *D* and *E* were under the defect.

**Figure 4:**
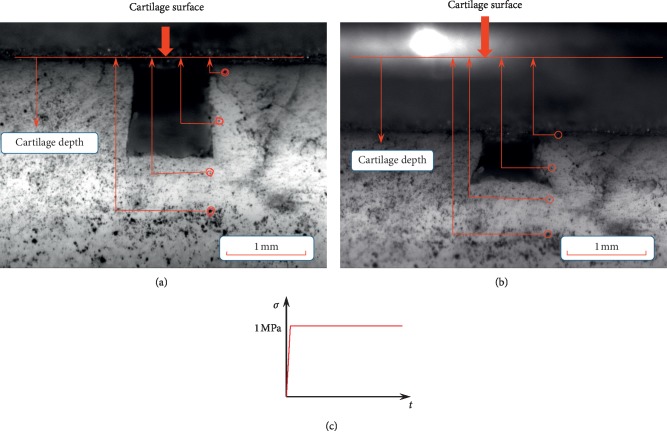
Microscopic images of articular cartilage. (a) Before load. (b) After load. (c) The schematic diagram of loading curve.

With the help of optimized DIC technique, the deformation of different layers for cartilage can be obtained by analyzing the location changes of mark points.  Figure 4 showed the position of mark points before and after deformation.

## 3. Results

**Figure 5:**
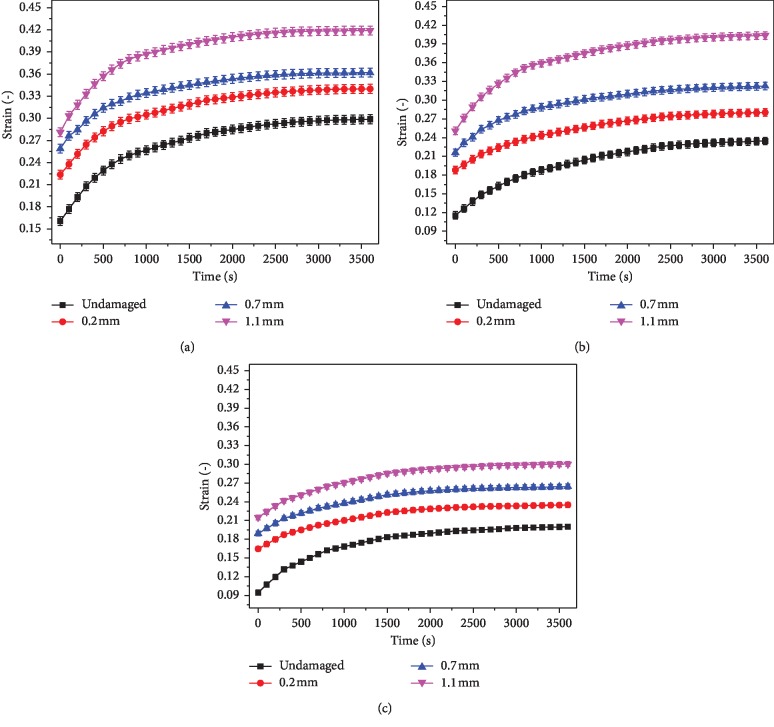
Creep curves of reference points near the defect in different layers of cartilage under the 1 MPa load. (a) Superficial layer. (b) Middle layer. (c) Deep layer.

The result data are obtained by analyzing the displacement of the reference points. Data points shown in the figure of this section represent mean value, whereas error bars indicate the standard errors above and below corresponding mean values.  Figure 5 showed the creep curve of the reference points in the different layers of cartilage under 1 MPa stress. It could be seen that the creep curve changes exponentially. The creep strains of reference points near the defect in the different layers increased rapidly in 500 seconds, and then the creep strains rose slowly. The creep strains of different layers near the cartilage defect increased gradually with the increase of defect depth. The creep in the each layer of cartilage basically reached balance at about 3000 seconds.

The creep strains in the superficial, medium, and deep layers of the intact cartilage were approximately 30%, 23%, and 20%, respectively. However, the creep strains of each layer in the cartilage with superficial defect were about 34%, 28%, and 23%, respectively. The creep strains of each layer were about 36%, 32%, and 26%, respectively, when the damage reached the medium layer. If the depth of defect reached the deep layer, the strains of each layer were about 41%, 40%, and 30%.

**Figure 6:**
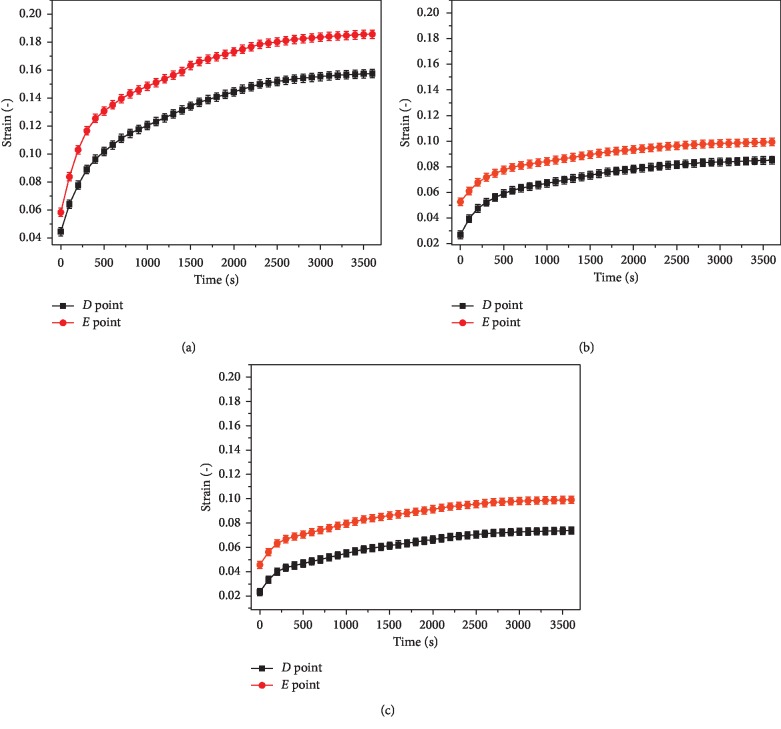
Creep curves of various reference points at the bottom of the cartilage defect under the condition of different defect depths. (a) 0.2 mm. (b) 0.7 mm. (c) 1.1 mm.

Figure 6 showed the creep changes of the points *D* and *E* at the bottom of the defect when the depth of damage reached 0.2 mm, 0.7 mm, and 1.1 mm, respectively. As could be seen from the figure, due to the stress concentration near the defect, the strain values at the point *E* were always greater than those at the point *D*. When the damage reached the superficial, middle, and deep layers, the strains at point *E* was about 18.5%, 10%, and 9.5%, respectively, and the strains at point *D* was about 15.5%, 8.5%, and 7%, respectively.

## 4. Creep Equation

Based on the mechanical behavior of viscoelastic materials, a three-parameter Kelvin model was used to describe its creep properties [14–16].

The general expression of the creep equation is as follows:

(1) $$\begin{array}{c} \varepsilon \left(t\right)=\frac{\sigma_{0}}{E_{1}}+\frac{\sigma_{0}}{E_{2}}\left[1-\exp\left(-\frac{t}{\tau }\right)\right], \end{array}$$

where *ε* is the creep strain value, *E*_1_ is the elastic modulus at the initial state, *E*_2_ is the elastic modulus at the steady state, *σ*_0_ is the applied constant stress, and the time constant *τ* is calculated by the least squares method. The creep equation parameters of cartilage with different depths of defects are shown in Table 1.

**Figure 7:**
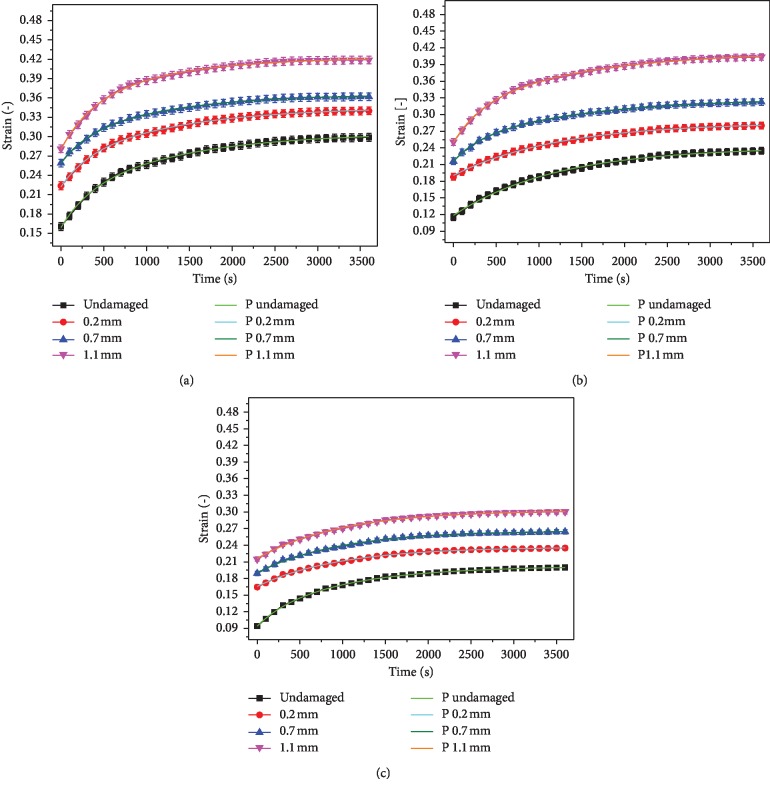
Comparison of predicted and experimental values of the creep equation. (a) Superficial layer. (b) Middle layer. (c) Deep layer.

Figure 7 shows a comparison of the creep equation with the experimental values. It could be seen that the predicted values of the creep equation agreed well with the experimental values, indicating that the established creep equation could describe the creep properties of articular cartilage.

## 5. Discussion

Creep experiment is an important method to study the viscoelastic behavior of articular cartilage. In this paper, the effect of microdefect on the creep behavior of articular cartilage was studied by the digital image correlation technology, and the three-parameter creep equation of articular cartilage was established. This research provides a reference for the establishment of numerical analysis model, and it will also provide a basis for studying the biomechanical mechanism of osteoarthritis development due to microdamage.

Experiments had shown that the creep curve of articular cartilage changed exponentially. Because of the microstructure features of cartilage, the creep curves of different layers were not coincident for the cartilage sample, and the creep strain decreased with the increase of cartilage depth. The creep trends in different layers were consistent with the results of Gao et al. [8, 9]. The creep strain near the microdefect still changed exponentially. The creep strain value of different layers near the defect area increased with the increase of damage depth. The defect had the greatest influence on the creep deformation of the superficial layer. Work of Grenier et al. [17] also found that the creep deformation of damaged articular cartilage was more than that of intact cartilage. It is the main reason that cartilage is a porous viscoelastic material, in which 80% is liquid and bearing load mostly is supported by the liquid. The interstitial fluid pressurization played a fundamental role in cartilage mechanics [18]. The nonlinear interaction between the hydraulic permeability of the cartilage and compressive strain on the cartilage retarded the progress of the consolidation of the cartilage during the compression [19]. At the initial stage of bearing load, there was the pressure difference between the inside and outside of the cartilage, and the liquid flowed out rapidly, resulting in a rapid change in the creep response of the cartilage. As the liquid flowed out continuously, the principal bearing part depending on the liquid was transformed into the solid, which indicated that the creep strain increased slowly until the creep curve reached equilibrium. When the cartilage was slightly damaged, the creep strain near the defect increased. On the one hand, the stress concentration near the defect increased pressure difference between the internal and external, resulting in the rapid flow of the liquid; on the other hand, structural damage reduced the bearing capacity of the cartilage in this region, resulting in greater shear deformation. The experiments had fully proved that the defect affected the creep property of cartilage and the mechanical microenvironment of chondrocytes. The curve in Figure 6 indicated that due to the stress concentration, the creep value at the point *E* was greater than that at other locations at the bottom. The three-parameter Kelvin model has been adopted by many scholars to describe the creep behavior of materials [14–16]. In this paper, the creep equation of articular cartilage was established by this model. The curves were found to be highly consistent with the creep curves obtained from the experiments. It is indicated that the creep equation established by the three-parameter model could describe the creep properties of the cartilage.

The experimental errors were principally derived from the position selection and location measurement of reference point. The samples came from different individual animals, which could also cause experimental errors. In these creep experiments, the animals were of the same age, and errors caused by the sample source did not change the creep trend. In order to reduce the error, each experimental sample was operated twice. Data points shown in the figure of result section represented mean values. The interval time between twice tests was set 2 hours in creep experiments. It was enough for the loaded cartilage to recover the initial states. The cartilage samples were dehydrated without destroying the tissue structure under 1 MPa load in the creep experiments. Boettcher et al. [20] found that articular cartilage could recover a broad range of its material properties after dehydration and rehydration provided that a physiological salt solution was used for rehydration. Although minor alterations in the microarchitecture of rehydrated cartilage in the superficial zone were detected, these alterations did not interfere with the viscoelastic and tribological properties of the tissue. The defect width of the sample in the experiments is much larger than the actual damage size. It is beneficial to magnify and study the effect of injury on the creep property of cartilage.

Some researchers studied the creep deformation of diseased cartilage [5, 6]. By comparative analysis, it was also found that the damaged cartilage and the diseased cartilage had different creep characteristics. The creep strain of the cartilage experienced mechanical defect was greater than the normal cartilage, while the pathological cartilage had reduced creep strains. Maybe the cartilages in our experiments were mechanically damaged and had not undergone tissue degeneration in a short period of time, while the tissue of the diseased cartilage in the literature had been denatured, which changed the mechanical properties [21, 22].

## 6. Conclusions

In this paper, creep experiments were performed on porcine articular cartilage with different depths defect. Compared with the intact healthy cartilage, the effects of defect depth on mechanical properties of articular cartilage were obtained and the creep equation was established. The conclusions of this study are as follows:

1. The creep of various layers in the articular cartilage changed exponentially, and the creep strain decreased with the increase of cartilage depth.
2. After mechanical damage, the creep deformation of articular cartilage in each layer still changed exponentially. As the depth of damage increased, the creep strain of each layer near the defect increased.
3. The creep strain of articular cartilage after mechanical initial damage was different from the creep strain of the diseased cartilage. The former had increased creep strain and the latter had reduced creep strain.
4. The three-parameter viscoelastic constitutive equation could predict the creep performance of articular cartilage well.

## Figures and Tables

**Table 1:** Parameters of the creep equation under the condition of different damage depths.

Location	Damage depth (mm)	*E*_1_ (MPa)	*E*_2_ (MPa)	Time constant *τ*
Point A (surface layer)	0	6.23	7.24	888
	0.2	4.47	8.61	873
	0.7	3.86	9.7	770
	1.1	3.86	7.24	770

Point B (middle layer)	0	8.66	8.4	1185
	0.2	5.32	10.83	1210
	0.7	4.63	9.39	975
	1.1	3.99	6.55	898

Point C (deep layer)	0	10.59	9.18	922
	0.2	6.08	14.21	994
	0.7	5.29	13.21	994
	1.1	4.65	11.68	1009

## Data Availability

The experimental results data used to support the findings of this study are included within the article. The experiment data supporting this analysis are from previously reported studies and data sets, which have been cited.

## References

[1] Mow V. C., Kuei S. C., Lai W. M., Armstrong C. G. (1980). Biphasic creep and stress relaxation of articular cartilage in compression: theory and experiments. *Journal of Biomechanical Engineering*.

[2] Li L. P., Korhonen R. K., Iivarinen J., Jurvelin J. S., Herzog W. (2008). Fluid pressure driven fibril reinforcement in creep and relaxation tests of articular cartilage. *Medical Engineering & Physics*.

[3] Taffetani M., Gottardi R., Gastaldi D., Raiteri R., Vena P. (2014). Poroelastic response of articular cartilage by nanoindentation creep tests at different characteristic lengths. *Medical Engineering & Physics*.

[4] Ikeuchi K., Oka M., Kubo S. (1994). The relation between friction and creep deformation in articular cartilage. *Tribology Series*.

[5] Fick J. M., Espino D. M. (2011). Articular cartilage surface rupture during compression: investigating the effects of tissue hydration in relation to matrix health. *Journal of the Mechanical Behavior of Biomedical Materials*.

[6] Cooke M. E., Lawless B. M., Jones S. W., Grover L. M. (2018). Matrix degradation in osteoarthritis primes the superficial region of cartilage for mechanical damage. *Acta Biomaterialia*.

[7] Chahine N. O., Wang C. C.-B., Hung C. T., Ateshian G. A. (2004). Anisotropic strain-dependent material properties of bovine articular cartilage in the transitional range from tension to compression. *Journal of Biomechanics*.

[8] Gao L.-L., Zhang C.-Q., Gao H., Liu Z.-D., Xiao P.-P. (2014). Depth and rate dependent mechanical behaviors for articular cartilage: experiments and theoretical predictions. *Materials Science and Engineering: C*.

[9] Gao L., Liu D., Gao H., Lv L., Zhang C. (2019). Effects of creep and creep-recovery on ratcheting strain of articular cartilage under cyclic compression. *Materials Science and Engineering: C*.

[10] Kim W., Thambyah A., Broom N. (2012). Does prior sustained compression make cartilage-on-bone more vulnerable to trauma?. *Clinical Biomechanics*.

[11] Zhang G., Turley S., Thambyah A., Broom N. D. With prior creep loading cartilage is more vulnerable to micro-level impact-induced failure.

[12] Thambyah A., Zhang G., Kim W., Broom N. D. (2012). Impact induced failure of cartilage-on-bone following creep loading: a microstructural and fracture mechanics study. *Journal of the Mechanical Behavior of Biomedical Materials*.

[13] Borrelli J., Zaegel M. A., Martinez M. D., Silva M. J. (2010). Diminished cartilage creep properties and increased trabecular bone density following a single, sub-fracture impact of the rabbit femoral condyle. *Journal of Orthopaedic Research*.

[14] Yang X., Cheng X., Luan Y., Liu Q., Zhang C. (2019). Creep experimental study on the lumbar intervertebral disk under vibration compression load. *Proceedings of the Institution of Mechanical Engineers, Part H: Journal of Engineering in Medicine*.

[15] Liu X., Yang C., Yu J. (2015). The influence of moisture content on the time-dependent characteristics of rock material and its application to the construction of a tunnel portal. *Advances in Materials Science and Engineering*.

[16] Haque M. N., Langrish T. A. G., Keep L.-B., Keey R. B. (2000). Model fitting for visco-elastic creep of Pinus radiata during kiln drying. *Wood Science and Technology*.

[17] Grenier S., Donnelly P. E., Gittens J., Torzilli P. A. (2015). Resurfacing damaged articular cartilage to restore compressive properties. *Journal of Biomechanics*.

[18] Soltz M. A., Ateshian G. A. (1998). Experimental verification and theoretical prediction of cartilage interstitial fluid pressurization at an impermeable contact interface in confined compression. *Journal of Biomechanics*.

[19] Mow V. C., Mansour J. M. (1977). The nonlinear interaction between cartilage deformation and interstitial fluid flow. *Journal of Biomechanics*.

[20] Boettcher K., Kienle S., Nachtsheim J., Burgkart R., Hugel T., Lieleg O. (2016). The structure and mechanical properties of articular cartilage are highly resilient towards transient dehydration. *Acta Biomaterialia*.

[21] Pritzker K. P. H., Gay S., Jimenez S. A. (2006). Osteoarthritis cartilage histopathology: grading and staging. *Osteoarthritis and Cartilage*.

[22] Ding C., Garnero P., Cicuttini F., Scott F., Cooley H., Jones G. (2005). Knee cartilage defects: association with early radiographic osteoarthritis, decreased cartilage volume, increased joint surface area and type II collagen breakdown. *Osteoarthritis and Cartilage*.

